# Effect of intranasal insulin administration on postoperative delirium prevention in elderly cardiac surgery patients: study protocol for a multicenter, double-blind, randomized, controlled trial

**DOI:** 10.1186/s13063-023-07860-6

**Published:** 2023-12-21

**Authors:** Yosuke Nakadate, Mariko Yamada, Natsuyo Kusuyama, Ryota Ishii, Hiroaki Sato, Thomas Schricker, Makoto Tanaka

**Affiliations:** 1 Department of Anesthesiology, University of Tsukuba Hospital, 2-1-1, Amakubo, Tsukuba, Ibaraki, 305-8576 Japan; 2 Department of Anesthesia, Tsukuba Medical Center Hospital, Ibaraki, Japan; 3 Department of Biostatistics, Faculty of Medicine, University of Tsukuba, Ibaraki, Japan; 4 Department of Anesthesia, McGill University Health Centre Glen Site, Royal Victoria Hospital, Montreal, Canada; 5 Department of Anesthesiology, Faculty of Medicine, University of Tsukuba, Ibaraki, Japan

**Keywords:** Intranasal insulin administration, Postoperative delirium, Cardiac surgery, Cardiopulmonary bypass

## Abstract

**Background:**

Postoperative delirium (POD) is a complication after surgery which leads to worse outcomes. The frequency of this syndrome is increasing as more elderly patients undergo major surgery. The frequency is around 10–25% but reaches as high as 50% for cardiac surgery. Although intranasal insulin (INI) administration of up to 160 units in patients with cognitive dysfunction and delirium has been shown to improve memory function and brain metabolism without complications such as hypoglycemia, it remains unknown whether INI prevents POD after cardiac surgery

**Methods:**

A multicenter, double-blind, randomized, controlled trial will be conducted at University of Tsukuba Hospital and Tsukuba Medical Center Hospital, Japan, from July 1, 2023, to December 31, 2025. A total of 110 elderly patients (65 years old or older) undergoing cardiac surgery requiring cardiopulmonary bypass will be enrolled and randomly allocated to intranasal insulin or intranasal saline groups. The primary outcome is the incidence of POD within 7 days after surgery. Secondary outcomes include days and times of delirium, screening tests of cognitive function, pain scores, duration of postoperative tracheal intubation, and length of ICU stay.

**Discussion:**

The present objective is to assess whether 80 IU INI administration during surgery prevents POD after cardiac surgery. The results may provide strategic choices to prevent POD in patients with cardiac surgery requiring cardiopulmonary bypass.

**Trial registration:**

The trial was registered with the Japan Registry for Clinical Trials with identifier jRCTs031230047 on April 21, 2023.

## Administrative information

Note: the numbers in curly brackets in this protocol refer to SPIRIT checklist item numbers. The order of the items has been modified to group similar items (see http://www.equator-network.org/reporting-guidelines/spirit-2013-statement-defining-standard-protocol-items-for-clinical-trials/).

**Table:** 

Title {1}	Effect of Intranasal Insulin Administration on Postoperative Delirium Prevention in Elderly Cardiac Surgery Patients: Study Protocol for A Multicenter, Double-Blind, Randomized, Controlled Trial
Trial registration {2a and 2b}.	jRCTs031230047 [Japan Registry for Clinical Trials] on April 21, 2023
Protocol version {3}	Version 1.0 (March 12, 2023)
Funding {4}	This study was supported by a Grant-in-Aid (C) for Scientific Research [KAKENHI Grant No. 23K08399] from the Japan Society for the Promotion of Science (JSPS) and Grant for Implementation of Advanced Medicine (GIAM) [Grant No. 110] from the University of Tsukuba Hospital.
Author details {5a}	Yosuke Nakadate^1^, Mariko Yamada^1^, Natsuyo Kusuyama^2^, Ryota Ishii^3^, Hiroaki Sato^4^, Thomas Schricker^4^, Makoto Tanaka^5^ 1. Department of Anesthesiology, University of Tsukuba Hospital 2. Department of Anesthesia, Tsukuba Medical Center Hospital 3. Department of Biostatistics, Faculty of Medicine, University of Tsukuba 4. Department of Anesthesia, McGill University Health Centre Glen Site, Royal Victoria Hospital 5. Department of Anesthesiology, Faculty of Medicine, University of Tsukuba
Name and contact information for the trial sponsor {5b}	Japan Society for the Promotion of Science (JSPS) 5-3-1 Kojimachi, Chiyoda, Tokyo, 102-0082, JAPAN Tel: +81-3-3263-4724 University of Tsukuba Hospital. 2-1-1 Amakubo, Tsukuba, Ibaraki, 305-8576, JAPAN Tel: +81-29-853-3900
Role of sponsor {5c}	The sponsors and funding bodies were not involved in the study design, collection, analysis, or interpretation of data; the writing of the report; or the decision to submit the article for publication.

## Introduction

### Background and rationale {6a}

Postoperative delirium (POD) is a complication after surgery in which acute disturbances of consciousness, attention, and perception appear and show diurnal fluctuations, resulting in impaired attention/thought processes and circadian rhythm disturbances [1]. This syndrome is increasing as more elderly patients undergo major surgery [2, 3]. The frequency is 10–25% [4, 5] but reaches as high as 50% for cardiac surgery [6].

Delirium is a short-term disease concept but has been shown to worsen long-term prognoses with increased lengths of hospital stay, postoperative complications, and mortality [7, 8]. Many patients with POD suffer mid-to-long-term postoperative neurocognitive dysfunction (cognitive dysfunction detected postoperatively in the absence of impaired consciousness using neuropsychological testing) [9], which leads to serious deteriorations in quality of life, such as an inability to work [10]. However, there is no standard prophylaxis for POD because pharmacological interventions, mainly anti-inflammatory, have not shown effective results in preventative treatment [11].

The precise mechanism of POD pathogenesis remains unknown but the neuroinflammatory response theory is the most likely explanation. Systemic inflammation induced by surgery and anesthesia causes acute brain inflammation [12]. In cardiac surgery, strong neuroinflammation is particularly induced by cardiopulmonary bypass (CPB), median sternotomy, myocardial ischemia-reperfusion injury, and various anesthetic agents [6], with these surgical factors thought to contribute to the high rate of delirium.

Intranasal insulin (INI) administration of up to 160 units in patients with cognitive dysfunction and delirium has been shown to improve memory function and brain metabolism [13–16] without hypoglycemia [17]. Glucose-insulin-normoglycemia therapy (high-dose intravenous insulin) [18] preserves short- and long-term memory after cardiac surgery [19] by suppressing inflammation in the brain due to insulin spilling over into the cerebrospinal fluid [20]. Insulin signaling in the brain was associated with cognitive function and perioperative inflammatory responses. Therefore, INI could be exploited to reduce POD and/or cognitive dysfunction.

### Objectives {7}

The objective is to determine whether preoperative INI administration reduces the incidence of POD in elderly patients undergoing cardiac surgery requiring cardiopulmonary bypass. In addition, the effects on cognitive function in the early postoperative period will be evaluated.

### Trial design {8}

This is a prospective, parallel-arm, double-blinded, randomized controlled trial. Consenting patients will be randomly assigned into the insulin or saline groups (1:1 allocation).

## Methods: participants, interventions, and outcomes

### Study setting {9}

The study will be conducted in the University of Tsukuba Hospital (academic hospital), Ibaraki, Japan, and Tsukuba Medical Center Hospital (tertiary hospital), Ibaraki, Japan.

### Eligibility criteria {10}

#### Inclusion criteria

1. Patients undergoing scheduled cardiac surgery using CPB
2. Patients who are 65 years of age or older at the time consent is obtained
3. Patients with no history of nasal surgery
4. Patients who have given informed, written consent for this clinical research

#### Exclusion criteria

1. Patients with preoperative delirium
2. Patients with a score of 23 or less on the preoperative Japanese version of mini-mental state examination (MMSE) [21, 22]
3. Patients scheduled to have hypothermia (32 degrees or lower) during surgery
4. Patients with a history of serious insulin allergy
5. Patients with severe psychiatric disorders (depression, bipolar disorder, schizophrenia, etc.)
6. Patients with obvious neurological diseases (Alzheimer’s disease, Parkinson’s disease, epilepsy, cerebral infarction, cerebral hemorrhage, etc.)
7. Patients with severe visual or hearing impairment
8. Patients who have difficulty communicating in Japanese
9. Patients deemed inappropriate by the principal or sub-investigators

### Who will take informed consent? {26a}

The research team members will invite eligible patients to participate in this study. The researchers (licensed medical doctors) will instruct them on the study protocol and obtain informed consent from all participants one to 60 days before surgery.

### Additional consent provisions for collection and use of participant data and biological specimens {26b}

Blood sampling (5 mL) will be performed just before surgery and at postoperative day 1, with samples preserved for a future study if patients consent to it.

## Interventions

### Explanation for the choice of comparators {6b}

INI may prevent POD because INI improves cognitive function in patients with cognitive impairment without causing hypoglycemia probably due to an anti-inflammatory effect described in the Introduction section.

There is currently no standard drug therapy to base protocols on, necessitating a de novo approach with established precedence from translational and clinical reports. As such, using placebo as a control is ethically acceptable.

### Intervention description {11a}

#### Anesthesia management

Medications on the day of surgery should be used only as necessary for anesthetic and perioperative management. In principle, hypoglycemic drugs should be discontinued but, if necessary, there are no restrictions on such medications, including diabetic drugs and drugs that may affect blood glucose levels (e.g., steroids),

Anesthesia care includes the use of the following monitors: a blood pressure monitor, electrocardiogram, oxygen saturation monitor, cerebral near-infrared spectroscopy monitoring and bispectral index monitor, and arterial catheter placed in the radial artery. After induction of anesthesia and tracheal intubation, a transesophageal echocardiography device will be inserted and a central venous catheter and pulmonary artery catheter will be placed through the right internal jugular vein.

Anesthesia methods are not specified and standard general anesthetics are to be used. In principle, extracellular fluid with 1% or less glucose content should be used as intravenous fluid but artificial colloid fluid, albumin, and blood products should be used as appropriate in case of decreased circulating plasma volume or hemorrhaging. Red blood cell transfusions will be used, if necessary, to achieve a hematocrit of at least 25%.

Before CPB, 300 IU/kg intravenous heparin should be administered followed by possible additional doses to keep the activating clotting time at 400–480 sec or longer. Protamine will be administered for heparin reversal after separation from CPB. During CPB, the pump flow rate should be 2.4 L/min/m^2^, the mean blood pressure should be 50–70 mmHg, and the body temperature should be maintained at 32–36 °C. Cardioplegia will be administered to initiate and maintain cardiac arrest.

A systolic blood pressure of 100 mmHg should be maintained before and after weaning from CPB with noradrenaline and dopamine used as needed.

If hypoglycemia (< 70 mg/dL) or hypoglycemia is strongly anticipated, a 20% glucose solution should be administered intravenously until normoglycemia is established. In principle, when blood glucose is >180 mg/dL, intravenous insulin will be administered to achieve a target blood glucose level of 70–180 mg/dL.

#### Study drug administration

After induction of anesthesia, the principal investigator or a staff investigator will administer the study drug intranasally into the right and left nasal cavities using a spray bottle.

##### Placebo group

After induction of anesthesia, the control drug (saline solution, Terumo Corp, Tokyo, Japan) will be administered into the left and right nasal cavities in 4 pushes each (0.1 mL per dose), for a total of 8 pushes.

##### Insulin 80 units group

After induction of anesthesia, the test drug (Humulin R, Eli Lilly Japan, Kobe, Japan) will be administered into the left and right nasal cavities in 4 pushes each (0.1 mL per dose), for a total of 8 pushes.

#### Criteria for discontinuing or modifying allocated interventions {11b}

1. When cardiac surgery is canceled or postponed and cannot be started within 60 days from the date of registration
2. Development or presence of severe preoperative laboratory abnormalities (renal dysfunction, hepatic dysfunction, etc.)
3. When it is found that the eligibility criteria are not met after enrollment
4. When the research enrollee withdraws consent
5. When it is difficult to continue the study due to adverse events, etc. within 7 days after surgery
6. When it is difficult to continue the study due to worsening of complications within 7 days after the surgery
7. When the investigator determines that discontinuation is appropriate for other reasons
8. When this clinical research as a whole is terminated

#### Strategies to improve adherence to interventions {11c}

Not applicable. Interventions in this study will be completed during general anesthesia and without the patients’ cooperation.

#### Relevant concomitant care permitted or prohibited during the trial {11d}

There are no restrictions regarding concomitant care during the trial.

#### Provisions for post-trial care {30}

All study participants receive standard care. This includes extended follow-up after study participation has ended, if deemed necessary by the treating physician. According to the provisions of the compensation agreement, the investigator will compensate the participant for physical damage caused by the clinical trial intervention through compensation insurance.

## Outcomes {12}

### Primary endpoint

Incidence of delirium within 7 days after surgery. Delirium will be evaluated for 7 days after cardiac surgery. During the intensive care unit (ICU) stay, the Confusion Assessment Methods for the Intensive Care Unit (CAM-ICU) [23, 24] will be conducted twice a day for each patient. In wards, the newly developed 3-min diagnostic assessment for delirium using the Confusion Assessment Method (3D-CAM) [25] will be conducted once a day for each patient.

A patient is considered to have delirium if he/she tests positive for delirium at least once during the postoperative period.

#### Secondary endpoint

1. Number of days with delirium
2. Number of times the patient has delirium
3. Change in MMSE-J between preoperative and 7 postoperative day
4. Change in the Japanese version of Montreal cognitive assessment (MoCA-J) [26, 27] between preoperative day 1 and postoperative day 7
5. Pain score numerical rating score (NRS) [28]
6. Length of postoperative tracheal intubation
7. Length of ICU stay

## Participant timeline {13}

**Fig. 1:**
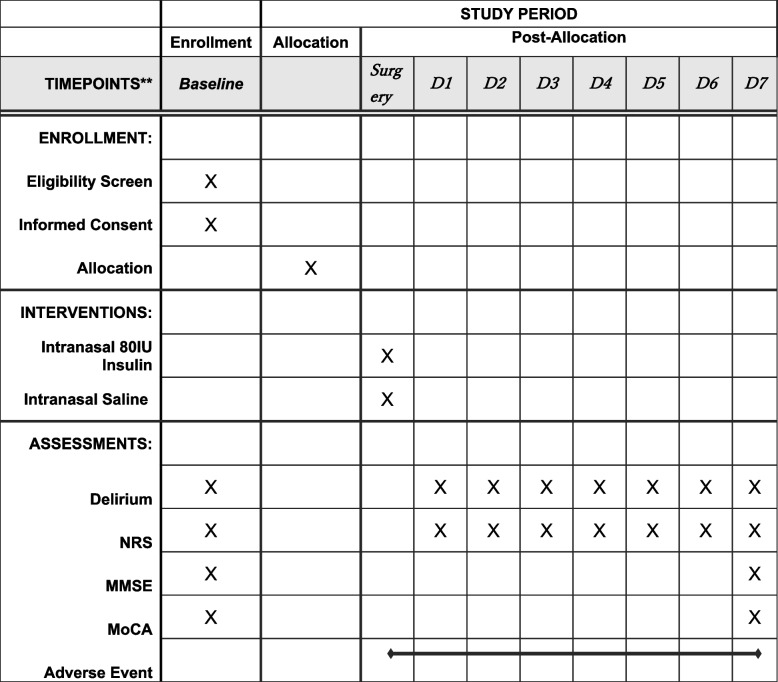
Participant timeline. D, postoperative day; MMSE, mini-mental state examination; NRS, numerical rating scale

The participant timeline is presented in Fig. 1.

### Sample size {14}

In a study conducted at the University of Yamanashi Hospital, 33% of patients were in the placebo group and 9.5% in the insulin group (unpublished data, jRCTs031200290). Although that previous study differs from the present study in that it excluded patients aged 20 years or older undergoing cardiac or major vascular surgery using cardiopulmonary ventilation and patients with severe renal dysfunction, the incidence of delirium seems reasonable.

Based on this, if the delirium rates in the placebo and insulin groups are 33% and 10%, respectively, with two-sided a significance level of 0.05 on both sides and a power of 0.8, the number of cases per group would be 49. We will recruit 55 cases per group for a total of 110 cases, taking into account 10% dropout cases.

### Recruitment {15}

Patients will be informed in detail of the course of the study, all study procedures, potential risks, and the benefits of each intervention 1–60 day(s) before surgery. Patients will be recruited at the University of Tsukuba Hospital and Tsukuba Medical Center Hospital.

## Assignment of interventions: allocation

### Sequence generation {16a}

Randomly assigned sequences will be generated using an electronic data capture (EDC) system (UHCT ACREeSS, https://plaza.umin.ac.jp/UHCTA/acress/). Eligible participants will be randomly assigned to the insulin or saline groups in a 1:1 ratio. Patients will be stratified and assigned to the University of Tsukuba Hospital and Tsukuba Medical Center Hospital.

### Concealment mechanism {16b}

During enrollment by the investigator, the EDC system (UHCT ACReSS, https://plaza.umin.ac.jp/UHCTA/acress/) will assign each participant a randomization number.

### Implementation {16c}

The pharmacy will hand out coded study medication (insulin or saline) to investigators before surgery. Concealment is ensured by a randomization list which is held by the pharmacy only. This list contains the randomization numbers and the assigned treatment groups.

## Assignment of interventions: blinding

### Who will be blinded {17a}

Trial participants, treating surgeons, and outcome assessors will be blinded to the treatment assignment. The principal investigator shall remain unblinded to detect and treat adverse events immediately. Therefore, the principal investigator shall not assess postoperative outcomes.

### Procedure for unblinding if needed {17b}

The pharmacy will hold the unblinding codes. In case of medical necessity or emergency related to the procedure, the principal investigator has the power to unblind and inform the patient and attending medical staff.

## Data collection and management

### Plans for assessment and collection of outcomes {18a}

Research data will be collected in an EDC system using ACReSS (UHCT ACREeSS, https://plaza.umin.ac.jp/UHCTA/acress/).

The following data will be collected:

Enrollment

1. Demographic data (sex, date of birth, race, height, weight, history of illness, medical history of primary illness, comorbidities, smoking history, medications, allergy history, previous surgical history, EuroScore II [29])
2. Laboratory and instrumental examinations
3. Delirium test: 3DCAM
4. Cognitive function screening tests (MMSE-J and MoCA-J)
5. Pain scale: NRS

During surgery

1. Blood glucose: Measured 20 min after study drug administration. If glucose trends downwards, measurement will be continued every 10 min and glucose solution will be administered if necessary until normoglycemia is achieved
2. Surgical data: Duration of surgery, anesthesia time, blood loss, volume of infusion, volume of blood transfused, urine output, minimum body temperature during CPB, duration of CPB, vasoactive drug use at discharge, fentanyl use, and remifentanil use

After surgery

1. Laboratory and instrument examinations
2. Delirium tests: CAM-ICU or 3DCAM
3. Cognitive function screening tests (MMSE-J and MoCA-J)
4. Adverse events
5. Pain scoring: NRS

### Plans to promote participant retention and complete follow-up {18b}

During the preoperative visit, researchers will explain the procedure of intraoperative management and postoperative follow-up in detail. The research team will conduct an assessment of the participants with careful consideration of their individual statuses.

### Data management {19}

An electronic clinical management system (ACReSS, University Hospital Clinical Trial Alliance, Chiba, Japan) will be used. Patient data are stored in raw medical records at each hospital and anonymized EDC. Research data is stored at the Department of Anesthesiology, University of Tsukuba Hospital, for 10 years after completion of the study. Changes in EDC are preserved with a log showing the information of who made changes and when.

### Confidentiality {27}

All patient data are anonymized in the EDC system. Only study investigators, who are given an original ID and password, may access the EDC and input data on patients at their facility.

### Plans for collection, laboratory evaluation and storage of biological specimens for genetic or molecular analysis in this trial/future use {33}

Blood sampling (5 mL) will be performed at just before surgery and postoperative day 1, with samples preserved for a future study if patients consent. The blood will be stored in a – 80 °C freezer at the Department of Anesthesia, University of Tsukuba Hospital.

## Statistical methods

### Statistical methods for primary and secondary outcomes {20a}

#### Primary endpoint

The incidence of delirium within the first postoperative week for each group will be determined. The ratio of the incidence rate in the insulin group versus the placebo group and its 95% confidence interval will be determined.

#### Patient background and secondary endpoints

Summary statistics will be obtained for each group. Continuous variables will be summarized by mean and standard deviation or median and interquartile range. Categorical variables will be summarized by frequency and proportion.

### Interim analyses {21b*}*

Not applicable. No interim analyses are to be performed because the study period is relatively short, the patient number is small, and the anticipated incidence of severe adverse events will be low. The study will be terminated (i) if it becomes impossible to achieve the planned number of patients due to difficulties in recruiting, (ii) if hypoglycemia occurs frequently in the insulin group, and/or (vi) if the research ethics board recommends study termination.

### Methods for additional analyses (e.g., subgroup analyses) {20b}

Not applicable. There are no subgroup analyses planned because the patient number is relatively small and most of the surgeries will be valve surgery.

### Methods in analysis to handle protocol non-adherence and any statistical methods to handle missing data {20c}

There will be no non-adherence issues in this study because the study drug is administered by the investigator. The possibility of missing values in the primary outcome will be very low because POD is assessed every day for seven days. Missing data will not be replaced.

### Plans to give access to the full protocol, participant-level data and statistical code {31c}

This study protocol is publicly available on https://jrct.niph.go.jp/en-latest-detail/jRCTs031230047. The datasets analyzed during the current study and statistical code are available from the corresponding author upon reasonable request, as is the full protocol.

## Oversight and monitoring

### Composition of the coordinating center and trial steering committee {5d}

The coordinating center is the Department of Anesthesiology, University of Tsukuba Hospital, Ibaraki, Japan. The trial steering committee consists of the principal investigator and sub-investigators. The principal investigator supervises and run day-to-day trial protocols and takes medical responsibility for patients. The investigators recruit patients and conduct the study and data entry. The data manager organizes data capture and safeguards quality and data. The drug control manager manages study drugs and dispenses them blinded to the investigators. The study team meets weekly.

The coordinating center team is supported daily by the Tsukuba Clinical Research and Development Organization (T-CReDO) of the University of Tsukuba Hospital, which is independent from the sponsor and competing interests.

The Certification of Clinical Trials Review Board, established at the University of Tsukuba Hospital as the trial steering committee, ensures that the study adheres to ethical principles and protects the patients’ health and dignity. It also checks the progress and completeness of this study. In case of any ethical violations, the study may be corrected or terminated.

### Composition of the data monitoring committee, its role and reporting structure {21a}

The monitoring manager is a member of the coordinating center team at the University of Tsukuba Hospital. On-site monitoring is performed at each hospital by monitors, who are not involved in interventions and evaluations for the patients, appointed by the monitoring committee. The preservation of consent forms, eligibility, outcomes, efficacy, and safety is the focus of each monitor. The monitoring practice is supported by the monitoring committee established by T-CReDO.

### Adverse event reporting and harms {22}

Severe adverse events are immediately reported to the principal investigator, who must report them to the Certification of Clinical Trials Review Board. Unexpected diseases that lead to death or potentially lead to death and that are suspected to be related to the study drugs must be reported to the Certification of Clinical Trials Review Board and Minister of Health, Labor and Welfare within 7 days. Expected diseases that lead to death or potentially lead to death and that are suspected to be related to the study drugs must be reported to the Certification of Clinical Trials Review Board. Unexpected diseases that lead to disability or potentially lead to disability or the continuation of a hospital stay and that are suspected to be related to the study drugs must be reported to the Certification of Clinical Trials Review Board and Minister of Health, Labor and Welfare within 15 days.

### Frequency and plans for auditing trial conduct {23}

Auditing will not be conducted for the present study. Protocol adherence and input data will be checked by monitoring. On-site monitoring will be performed at each hospital by monitors when the first five patients in each hospital are recruited and have all data captured then for every five to ten patients thereafter. The monitoring will also be performed before recruiting the first patient and after capturing all study patient data. Monitoring team meetings will be routinely held once a week. The monitoring team members will attend the monthly meetings of the monitoring committee established by T-CReDO.

### Plans for communicating important protocol amendments to relevant parties (e.g., trial participants, ethical committees) {25}

Any amendments to the protocol will be posted in the trial registry after approval by the Institutional Review Board.

### Dissemination plans {31a}

The results of this study will be presented at relevant international conference and published peer-reviewed journals. Both positive and negative results will be reported.

## Discussion

The current study is a randomized clinical trial evaluating intranasal insulin administration to reduce POD after surgery.

In animal models, prophylactic INI administration under isoflurane anesthesia in mice preserves memory function [30] and is mediated by insulin (mTOR-eEF2) signaling [31]. In abdominal surgery rat models, INI in aged rats on the day of, and for 3 days after surgery, remediated surgery-induced hippocampal neuroinflammation and hyperactivation of GSK-3β via insulin signaling, preventing cognitive impairment [32].

A clinical study on POD in patients undergoing laparoscopic radical surgery for gastrointestinal malignancies showed that 20 units of intranasal insulin administered for 2 days before surgery (5 times in total) significantly reduced delirium (12.5% in the insulin group vs. 47.5% in the placebo group) and suppressed systemic inflammation during the 5 days after surgery [33]. In older patients undergoing radical resection for esophageal cancer, 20 and 30 units of intranasal insulin administered for 2 days before surgery (5 times in total) also decreased POD prevalence within 3 days postoperatively (30% in the 20 unit insulin group and 3.3% in the 30 unit insulin group vs. 63.3% in the placebo group, *P* = 0.019 and *P* < 0.001, respectively) [34]. However, no clinical studies on cardiac surgery have been reported.

In the present study, we are focusing on vulnerable patients for POD. We will include relatively older patients undergoing cardiac surgery requiring CPB because age and cardiac surgery are identifiable and significant risk factors, especially since CPB causes strong inflammation [5, 6].

However, we will exclude cases receiving hypothermia, which means predominantly major vascular surgeries will be excluded to equalize the basic surgical procedures (i.e., pre- and post-CPB, plus CPB circulation) since such surgeries need selective cerebral perfusion during CPB and induce higher risks of stroke [35].

Our outcomes are incidence of delirium and cognitive function. Therefore, we will exclude patients with preoperative delirium, low MMSE scores, and those with neurological diseases to equalize baseline consciousness and cognitive function. We also will exclude patients with psychiatric disorders, visual or hearing impairments, or difficulty communicating in Japanese to avoid confusion and confounding of cognitive evaluations.

We have some limitations. First, delirium will only be assessed twice a day using the CAM-ICU in the ICU. We may thus overlook delirium because the specificity is low. Also, as we exclude patients with preoperative delirium, we cannot evaluate the therapeutic effect of INI. Nevertheless, we believe our protocol to be robust enough to evaluate the effect of INI for cardiac surgery patients, who are the most at-risk demographic for POD. If the effects of INI on POD and cognitive function are clarified, this could become a key treatment strategy for avoiding perioperative neurocognitive disorders, including delirium.

## Trial status

At the time of manuscript submission, the study is in the preparation phase for recruitment. This is protocol version 1.0. completed on March 12, 2023. Recruitment is scheduled to begin in July 2023 and expected to be completed in March 2025.

## Abbreviations

CAM-ICU: Confusion assessment methods for intensive care unit
CPB: Cardiopulmonary bypass
EDC: Electronic data capture
ICU: Intensive care unit
INI: Intranasal insulin
MMSE: Mini-mental state examination
MoCA: Montreal cognitive assessment
NRS: Numerical rating score
POD: Postoperative delirium
3D-CAM: 3-min diagnostic assessment for delirium using the confusion assessment methods
